# Visual art instruction in medical education: a narrative review

**DOI:** 10.1080/10872981.2018.1558657

**Published:** 2019-02-27

**Authors:** Neha Mukunda, Nazanin Moghbeli, Adam Rizzo, Suzannah Niepold, Barbara Bassett, Horace M. DeLisser

**Affiliations:** a Perelman School of School of Medicine, University of Pennsylvania, Philadelphia, PA, USA; b Division of Education, Philadelphia Museum of Art, Philadelphia, PA, USA

**Keywords:** Medical humanities, visual arts, observation skills, Visual Thinking Strategies, Artful Thinking

## Abstract

The humanities have been increasingly incorporated into medical school curricula in order to promote clinical skills and professional formation. To understand its current use, we reviewed the literature on visual arts training in medical education, including relevant qualitative and quantitative data. Common themes that emerged from this review included a focus on preclinical students; instruction promoting observation, diagnostic skills, empathy, team building, communication skills, resilience, and cultural sensitivity. Successful partnerships have involved local art museums, with sessions led primarily by art educators employing validated pedagogy such as Visual Thinking Strategies or Artful Thinking. There is evidence that structured visual arts curricula can facilitate the development of clinical observational skills, although these studies are limited in that they have been single-institution reports, short term, involved small numbers of students and often lacked controls. There is a paucity of rigorous published data demonstrating that medial student art education training promotes empathy, team building, communication skills, wellness and resilience, or cultural sensitivity. Given these concerns, recommendations are offered for fostering more robust, evidence-based approaches for using visual arts instruction in the training of medical students.

## Introduction

Over the last three decades, the humanities have been increasingly incorporated into medical school curricula [1]. This is a direct response to several acknowledged deficiencies in medical student education, including a lack of intentional training in clinical observation [2–4], the continued need for experiential approaches for promoting empathy [5–7], the disproportionate emphasis on technical over relational competence [4,8–10], and the rising need to foster trainee resilience and self-care [11,12]. In this regard, instruction in the humanities has been employed within medical education to build skills of observation, reflection, and introspection, flexibility in thinking, and tolerance for uncertainty and ambiguity [1].

The majority of initiatives employing humanities in student curricula have focused on training in the visual arts, with published literature reporting improvements in skills of observation, empathy, and resilience among medical students [13,14]. These studies, while promising, are limited in that they have been short term, anecdotal, and/or qualitative and descriptive [14]. In this paper, we will focus on visual arts training, reviewing existing pedagogy, representative programs, and illustrative published data, with the goal of providing a framework for future directions in this area of medical education.

## Overview of medical student visual arts education

### Research plan

We searched the literature in PubMed and Google Scholar databases for all publications about humanities and/or visual arts programs at accredited medical institutions. We then narrowed our search to medical institutions in the USA. From this search, we identified 11 institutions with detailed, publicly available curricula and seven institutions with published analyses, which we included in our review. Through analysis of the 11 programs’ syllabi, we designated five distinct ‘domains of focus,’ which allowed us to compare across institutions and systematically characterize the current scope of visual art instruction in medical education.

### Summary of existing programs

According to an updated report from ‘The Art of Examination: Art Museum and Medical School Partnerships Forum’ from May 2017 [15], there are nearly 70 medical schools in the USA as well as in Canada, Australia, and Italy, that are offering courses in the arts for their students. The overwhelming majority of the these are offered as elective courses, with only four being required components of the curriculum [16]. For this review, we focused on 11 medical schools in the USA (Table 1) whose visual arts programs/courses are particularly well structured and expansive [16]. As summarized in Table 1, these courses typically target preclinical medical students; involve partnerships with local museums; and incorporate a variable number of sessions, ranging from 4 to 18, which ranged from 1.5 to 2.5 h in length.

**Table 1.** Summary of selected programs.

School, program name	Targeted audience	Museum/community partnership	Session number	Session duration (hours)
Baylor College of Medicine, ‘Art of the Human Body’	Medical Students, year unspecified	Houston Museum of Fine Arts	4	2
Columbia University Vagelos College of Physicians and Surgeons, Weill Cornell Medical College, ‘Observation and Uncertainty in Art and Medicine’	MS1	Metropolitan Museum of Art, New York City	6	2
Harvard Medical School, ‘Training the Eye: Improving the Art of Physical Diagnosis’	MS1	Museum of Fine Arts, Boston	9	2.5
Icahn School of Medicine, ‘The Pulse of Art’	MS1	Solomon R. Guggenheim Museum	11	1.5
Perelman School of Medicine, ‘Art, Observation, and Empathy’	MS1	Philadelphia Museum of Art	7	1.5
Rush Medical College, ‘Humanities in Medicine’	MS1	The Art Institute of Chicago	18	?
University of Buffalo, ‘Learning to Look: An Artist’s Remedy to the Physician’s Perspective’	MS1-MS3 (3-year curriculum)	Expressive Visual Arts program for adult and adolescent inpatients, Aspire of WNY, Inc., Aspire Center for Learning	8	2
University of Cincinnati, ‘Art of the Clinical Encounter’	Medical Students, year unspecified	Cincinnati Art Museum	6	1.5
University of Washington, ‘Visual Thinking: How to Observe in Depth’	MS1, MS2	Henry Art Gallery, Seattle Art Museum, Frye Art Museum	10	1.5
UT Health San Antonio, ‘Art Rounds’	Interprofessional (nursing and medical students)	McNay Art Museum	8	1.5
UT Southwestern, ‘The Art of Examination’	MS1, MS2	Dallas Museum of Art, Nasher Sculpture Center, The Warehouse, The Crow Collection of Asian Art	7	2

MS1, MS2, and MS3 are respectively, medical student years 1, 2, and 3.

Five domains of focus were identified within these visual arts programs: (1) observation and diagnosis; (2) empathy; (3) team building/communication; (4) wellness/resilience; and (5) cultural sensitivity (Table 2).

**Table 2.** Key components to visual arts in medicine courses. Five categories were identified (clinical observation/diagnosis, empathy, team building/communication, promoting wellness/preventing burnout, and cultural sensitivity). Several examples are presented here, along with illustrative activities and relevant goals/objectives pertaining to each one.

Category	Examples of schools	Example of relevant activity or goal/objective
**Clinical observation/diagnosis**	Harvard Medical School	Paired guided museum visits (based on VTS) with clinical lectures to establish correlations with observational skills and diagnosis/physical exam
	UT Health Science Center, San Antonio	Students choose ‘Art Patient’ at the beginning of the course, and visit the artwork for at least 20 min in observation each time they are at the museum; ‘Differential Diagnosis’ assignment where students observe a portrait and assign possible medical diagnoses based on evidence
	UT Southwestern	‘Drawers and Describers’ – one student describes portrait and the other draws, followed by discussion of challenges of physical exam
**Empathy**	Baylor College of Medicine	*‘*Demonstrate empathetic communication in the discussion of the human body’
	Perelman School of Medicine	*‘*Foster cognitive skills that are the basis of empathy’
	Rush Medical College	*‘*Use artwork as a tool to build and consider empathy in medical practice’
	University of Cincinnati	Consecutive sessions on interpretation, mood, emotional response, and reflection, guiding students to understand the psychological mood evoked by an artist and the students’ emotional reaction
**Team building/communication**	Perelman School of Medicine	Students share individual reflections each week to foster trust and collaborative communication; ‘Back-to-Back Describe and Draw’ activity with partners
	Baylor College of Medicine	Session on ‘Constructing Narrative’ where students work together to interpret art and construct a collaborative story
	Columbia University Vagelos College of Physicians and Surgeons, Weill Cornell Medical College	‘Verbal Imaging’ – one student is ‘blinded’ and told to create a mental image while other students describe a piece of artwork in detail. Student then looks at painting to compare mental visualization with actual image
**Promoting wellness/preventing burnout**	University of Buffalo	‘To learn how art can enhance one’s personal growth as a physician’
	Rush Medical College	*‘*Examine self-care and its effect on practice’; ‘Play through dance and movement’
	UT Southwestern	Guided mindfulness, journal assignment on self-care
**Cultural sensitivity**	Icahn School of Medicine	*‘*Increase their respect of the differences among colleagues and among the people who need medical attention’
	University of Cincinnati	‘Identify one’s own biases and perceptions as integral elements of interpretation through examination of the cultural, ethnic, age and gender context of subjects depicted in photographs and portraiture.’
	Baylor College of Medicine	Session on ‘Understanding Bias,’ discussing depictions of the human form in art and compares cross-cultural art forms to identify ideals and biases
	Perelman School of Medicine	Weeks 5 and 6 involve ‘Perspective Taking’, where students imagine artwork from various perspectives and create narratives for the people in artwork and photographs. Educators choose artwork from multiple cultures and artwork that depicts interactions between people of different backgrounds

While most schools’ programs incorporated some aspect of all five domains, the specific content and pedagogy were varied (Table 3). Approaches employed to develop observational and diagnostic skills include guided art observation (most often conducted at an art museum), lectures on clinical correlations, focused observational sketches and patient interactions. Empathy was most often fostered through exercises that promote active recognition of emotion in artwork and mindfulness of one’s own emotional response. The inclusion of frequent small and large group discussions and team narrative building is a common feature of these courses that facilitates the development of trust between students to promote positive team dynamics and support relational communication. Wellness and resilience are promoted through activities including regular individual journaling, drawing exercises, and encouragement of mindfulness through art observation. Finally, programs addressed cultural sensitivity by exercises that encourage students to share various perspectives on a given piece of art, explore the cultural context of an artist’s work, and openly acknowledge and challenge predetermined biases.

**Table 3.** Selected program components of interest with examples of each.

Program component	Examples of schools	Descriptions
**Direct clinical correlation**	Harvard Medical School	Clinical lectures focusing on visual diagnosis and physical exam from various specialties (Radiology, Dermatology, Neurology); clinical Rounds where students examine patients with course faculty
	University of Buffalo	“Art Activity Clinical Experience Sites”; Standardized Patient Post-Assessment
	University of Cincinnati	Students attend one monthly half day session with faculty, where they initially record patient descriptions, and work towards full describe/interpret/reflect by course end
	Icahn School of Medicine	‘Skin Deep’ – students paint dermatologic conditions in own skin shade
**Interprofessional**	UT Health Science Center, San Antonio	Collaboration between nursing, medical, and school of allied health. Students from different disciplines are paired for ‘Art Patient’ activity and are required to complete assignments as a team
**Historical context**	Icahn School of Medicine	Sessions including ‘Picturing Pandemic Disease: From Plague to Ebola’; ‘*The Gross Clinic* by Thomas Eakins: The Ascendancy of American Medicine in the Nineteenth Century’
**Art forms outside of fine/visual arts**	Rush Medical College	Use of theater to explore role of body language, movement, and enacting role of patient and doctor on stage; Building auditory skills to explore how vocal sounds reveal emotion
**Assignments**	Various Schools	Suggested vs. mandatory weekly readings/videos (articles, Ted Talks), written/drawn reflections, clinical descriptions/sketches of patients, attending additional museum programs, final presentation
**Drawing exercises**	Harvard Medical School	Sketchbook journal given to all students at the beginning of the course to encourage habitual drawing, ongoing reflection, and connections between art and the physical exam
	University of Buffalo	Paired ‘studio session’ with each class – activities include self-portrait drawings, paint and dye on cloth/fabric, figure drawing, and clay figure sculpting
	Columbia University Vagelos College of Physicians and Surgeons, Weill Cornell Medical College, ‘Observation and Uncertainty in Art and Medicine’	Sketch memories, associations, and/or emotions associated with observing artwork ‘Sketching in the round’ – students sit in circle, sketch for 2 min, then rotate and pick up sketch where previous student left off to challenge the singularity of their perspective

### Pedagogy for teaching observation

Two approaches, Visual Thinking Strategies (VTS) [17–20] and Artful Thinking [2,20], have been typically employed in teaching the observational component of these programs.

VTS [17–19] was originally designed by psychologist Abigail Housen and art educator Philip Yenawine to help students learn to use visual arts to build observation and critical thinking skills [17]. It is based on three questions: (1) ‘What is going on in this picture?’ (2) ‘What do you see that makes you say that?’ and 3) ‘What more can you find?’ Together, these questions encourage students to look closely at art, make observations based on what they see, and share in collaborative meaning-making. Teachers and facilitators are encouraged to listen closely, point out what students are referencing, paraphrase, and encourage multiple responses to their prompts. Due to its validation as well as applicability to building observational skills, this pedagogy has been adopted by numerous medical schools in the design of their art curriculum [20].

Artful Thinking is a similar framework that has been utilized in medical school art programs [2]. It was developed by Project Zero with the goals of helping teachers build connections between curricular material and art and subsequently integrate art and music into their classrooms [21]. The program achieves this through simple ‘thinking routines,’ or short interactive activities that encourage close observation. Each of these routines is designed to build one of six specific ‘thinking dispositions’ in students, including reasoning, questioning and investigating, observing and describing, comparing and connecting, finding complexity, and exploring viewpoints. In addition, in contrast to VTS, contextual information is included to further facilitate the processing of the routines. The simple, targeted, and generalizable routines of Artful Thinking have been used broadly in various museum and post-secondary settings to facilitate learning through art observation [21,22].

### Museum/medical school partnerships

Partnerships between the medical school and a local art museum have been central to almost all of these programs (Table 1). This allows for a utilization of existing expertise from art educators that ensures a more effective learning experience for the students [23]. Most partnerships have been coordinated jointly by clinicians and museum educators, with the latter typically taking the lead on the instruction during individual sessions. However, depending on the course/program, clinicians and/or senior medical students will variably contribute to the session, enabling students to recognize and appreciate parallels between medicine’s core clinical skills and art observation.

## Educational impact of medical student visual arts education

Although subjective feedback is typically collected from participants in medical student art education courses/programs, there are few published detailed and/or rigorous reports on the educational impact of this training. Of these reports, studies from six institutions are summarized below.

### Yale University School of Medicine (2001)[24]

This course was offered to first year medical students starting their doctor–patient encounter course, and was named the ‘Yale Center for British Art (YCBA) Project’. From 1998 to 1999, 90 students were randomized into a control group (30 students), YCBA group (30 students), and lecture group (30 students). In the following year, 86 students were randomized to the YCBA group (51 students) or control group (35 students) after the lecture group showed no benefit. The control group went to clinical sessions led by a preceptor who taught the history and physical exam; the lecture group attended radiology lectures with imaging related to anatomy lab; and the YCBA group engaged in facilitated art observation [24].

The students received a pre- and post-test, in which they were given an image of patient and 3 min to write a detailed description, which were then blinded and scored for visual diagnostic elements. The results demonstrated a significant improvement in the post-test scores of the YCBA group for both years [24].

### University of Cincinnati Department of Family Medicine (2006)[25]

This qualitative study was unique in that it looked at the delayed effect of its course on the clinical phase of medical education. The 8-month elective course was initially offered to 19 second year medical students, who were then asked to participate during their third and fourth years after they had completed 9 or more months of clinical rotations. They were given eight questions and asked to provide subjective journal responses with specific examples, including ‘the most memorable experience, influence on and understanding biases in the doctor-patient relationship, influence on second year of medical school, usefulness during clinical years of medical school, unique skills or insights, and influence on experiencing art-related activities.’ [25]

Analysis of the responses demonstrated that (1) visual arts alongside an integrated clinical component was necessary for improving observational skills, (2) description and interpretation are not systematically taught in other areas of the medical curriculum, and (3) that medical students felt that time for reflection was critical to their education and personal growth [25].

### Harvard Medical School (2008)[4]

‘Training the Eye: Improving the Art of Physical Diagnosis’ is a multidisciplinary course that aims to improve visual literacy skills by practicing ‘unbiased inspection and accurate reporting.’ [4] It consists of seven sessions, each with 75 min of observation at the Boston Museum of Fine Art followed by a 1-h lecture relating visual arts with physical diagnosis. There is also an optional session for students to draw a live model with professional art instruction.

The study was a prospective, partially randomized pre- vs. post-course evaluation. There were 24 students in the intervention group, and 34 in the control group. The pre- and post-tests were distinct 1-h visual skills examinations in which students were asked to describe three patients with various visual diagnoses and two works of fine art.

The results demonstrated a consistent increase in post-test scores in students who took the course as compared to the control group. The study also found a possible graded effect of class attendance on post-test scores, which may advocate for continued practice throughout medical training. Additionally, qualitative analysis showed a significant improvement in five response categories of the intervention group which were determined to have clinical correlates: observations; interpretation supported by evidence; speculative thinking; pertinent negatives; and use of fine arts concepts [4].

### Robert Wood Johnson Medical School (2013)[3]

Robert Wood Johnson Medical School conducted a non-randomized, non-controlled qualitative study on one 3-h long session during a required week ‘Introduction to the Clerkship Experience,’ held for all third year medical students prior to clerkships. There was no museum partnership; instead, students were guided by a fourth year medical student through a large group VTS-based discussion on eight representational and non-representational fine art pieces.

Students were assessed with a pre- and post-test, during which they were asked to list observations and give a detailed description of two photographs of patients with visible medical conditions. While the study found no significant difference in the number of observations between pre- and post-tests, analysis of the descriptions revealed significant improvement in four clinically relevant themes: use of subjective terminology; scope of interpretations; speculative thinking; and use of visual analogies. The authors concluded that the session is an easily generalizable intervention with an appreciable impact on clinical observational skills.

### University of Pennsylvania, Perelman School of Medicine (2018)[2]

This randomized, single-masked control trial enrolled 36 first year medical students at the Perelman School of Medicine and randomized them 1:1 into either the control or intervention group. The intervention group received free museum passes and attended art observation sessions led by art educators at the Philadelphia Museum of Art, while the control group only received free passes. The sessions were designed using the ‘Artful Thinking’ approach; the curriculum was based entirely on art observation without any formal clinical or lecture component.

The analysis consisted of a computerized pre- and post-test in which students were asked to describe art images, retinal photographs, and clinical photographs involving ocular surface/peri-ocular disease. These descriptions were then scored by graders who were masked to both group (control or intervention) and to time (pre/post). Clinical educators scored the retinal and clinical photographs based on a predetermined a-priori rubric, while the museum educators scored the art images based on how often students described themes of ‘Observing, Interpreting, Evaluating, Associating, Problem-Finding, Comparing, Flexible Thinking, and Evidence.’ [2]

The results demonstrated significantly increased scores between the pre- and post-tests in students in the intervention group, and a decrease in scores in the control group. This increase in scores occurred without any formal medical clinical correlation component, to which the authors concluded that art training in isolation can help teach medical students to become better clinical observers. Finally, the study also analyzed emotional recognition test scores, which did show a trend toward improvement in the intervention group.

### Weill Cornell Medical College and Columbia University Vagelos College of Physicians and Surgeons (2018) [26]

‘Observation and Uncertainty in Art and Medicine’ was designed to improve tolerance of uncertainty and reflective capacity through art observation. It represents a unique collaborative effort between Weill Cornell and Columbia University and enrolls six students per year from both institutions.

The published analysis involves 4 years of course administration with a total of 47 student participants. The researchers employed a mixed-methods analysis involving (1) quantitative analysis of three pre- and post-course surveys aimed at studying the course objectives (Groningen Reflection Ability Scale (GRAS); Tolerance for Ambiguity (TFA) scale; and Best Intentions Questionnaire (BIQ) for awareness of personal bias) and (2) qualitative analysis of focus group transcripts and written evaluations.

Results demonstrated a statistically significant improvement in GRAS scores after completion of the course, indicating improvement in student reflection. There was also a small improvement in TFA and BIQ scores, although these did not show statistical significance. The authors identified four major themes of improvement from the qualitative evaluations, including ‘enhancement of observational skills, awareness of the subjectivity and uncertainty of perception, exploration of multiple points of view, and recognition of the course as a place for restoration and connection to classmates.’

## Discussion and recommendations

From this review, common course themes for medical student visual arts education (Tables 1 and 2) included a focus on preclinical students; instruction promoting the domains of observation and diagnostic skills, empathy, team building/communication skills, wellness/resilience, and cultural sensitivity; and content and activities enabling the mutual sharing perspectives, collaborative meaning-making, individual reflection time, and direct challenging of biases. Successful partnerships have involved local art museums, with sessions led primarily by museum educators who employ existing validated pedagogy such as VTS or Artful Thinking.

The studies described above provide reasonable evidence that structured visual arts curricula can facilitate the development of clinical observational skills. In contrast, although anecdotal experience and trends in some reports suggest a benefit, there is a paucity of rigorous published data demonstrating that medial student art education training promotes empathy, team building, communication skills, wellness/resilience, or cultural sensitivity. In regard to the studies that have demonstrated an educational impact, they have all involved single institutions, with small cohorts lacking controls, and thus the generalizability of their findings is unknown. Further, these studies have not objectively accessed the durability of improvements in art observation or clinical skills and have not sorted out the ‘dose’ required for an effect. Additionally, there are unresolved issues about pedagogy. Further, although earlier studies suggested a direct benefit of integrated clinical components [4,25] in the art education training, a more recent study demonstrated a comparable increase in clinical observational skills with art observation training alone [2]. Consequently, the role of integrating explicit clinical training with the art observation training requires further exploration.

Given the concerns noted above, it is clear there is a need for more robust, evidence-based approaches for using visual arts instruction in the training of medical students. We therefore recommend the following:

1. Moving beyond assessments of student satisfaction to more rigorous, prospective assessments of course offerings
2. Pre- and post-course surveys and assessments, using validated measures, that explicitly assess the five areas potentially impacted by visual arts training
3. Inclusion of control groups and the use of randomization to remove confounding effects of instruction in other parts of the curriculum
4. Prospective evaluations of the amount of instruction (i.e., ‘dose’) required for effects and the durability of those effects
5. Providing course offerings that target post-clerkship students (with appropriate assessments)
6. Collaborative partnerships between medical schools (especially in areas with two or more medical schools in close proximity to each other, similar to the model established by Weill Cornell and Columbia University) to jointly develop and implement visual arts education offerings

We believe the implementation of these recommendations, building on recent efforts such as that of *The Art of Examination: Art Museum and Medical School Partnership Forum* [15,16], will help to spur scholarship that furthers the quality of visual arts instruction received by medical students.
